# Suppression of cell migration is promoted by miR-944 through targeting of SIAH1 and PTP4A1 in breast cancer cells

**DOI:** 10.1186/s12885-016-2470-3

**Published:** 2016-07-04

**Authors:** Ali Flores-Pérez, Laurence A. Marchat, Sergio Rodríguez-Cuevas, Verónica Piña Bautista, Lizeth Fuentes-Mera, Diana Romero-Zamora, Anabel Maciel-Dominguez, Olga Hernández de la Cruz, Miguel Fonseca-Sánchez, Erika Ruíz-García, Horacio Astudillo-de la Vega, César López-Camarillo

**Affiliations:** Universidad Autónoma de la Ciudad de México, Posgrado en Ciencias Genómicas, Ciudad de México, México; Programa en Biomedicina Molecular y Red de Biotecnología, Escuela Nacional de Medicina y Homeopatía, Instituto Politécnico Nacional, Ciudad de México, México; Instituto de Enfermedades de la Mama, FUCAM, Ciudad de México, México; Universidad Autónoma de Nuevo León, CIDICS, Ciudad de México, México; Laboratorio de Medicina Translacional, Instituto Nacional de Cancerología, Ciudad de México, México; Laboratorio de Investigación en Cáncer Translacional y Terapia Celular, Centro Médico Siglo XXI, Ciudad de México, México; San Lorenzo 290. Col. Del Valle. CP 03100, Mexico City, México

**Keywords:** Breast cancer, miR-944, Migration, Invasion, Actin cytoskeleton, SIAH1, PTP4A1

## Abstract

**Background:**

Aberrant expression of microRNAs has been associated with migration of tumor cells. In this study, we aimed to investigate the biological significance of miR-944 whose function is unknown in breast cancer.

**Methods:**

MiR-944 expression in breast cancer cells and tumors was evaluated by Taqman qRT-PCR assays. Transcriptional profiling of MDA-MB-231 cells expressing miR-944 was performed using DNA microarrays. Cell viability, migration and invasion were assessed by MTT, scratch/wound-healing and transwell chamber assays, respectively. The luciferase reporter assay was used to evaluate targeting of SIAH1, PTP4A1 and PRKCA genes by miR-944. SIAH1 protein levels were measured by Western blot. Silencing of SIAH1 gene was performed by RNA interference using shRNAs.

**Results:**

Our data showed that miR-944 expression was severely repressed in clinical specimens and breast cancer cell lines. Suppression of miR-944 levels was independent of hormonal status and metastatic potential of breast cancer cells. Gain-of-function analysis indicated that miR-944 altered the actin cytoskeleton dynamics and impaired cell migration and invasion. Genome-wide transcriptional profiling of MDA-MB-231 cells that ectopically express miR-944 showed that 15 genes involved in migration were significantly repressed. Notably, luciferase reporter assays confirmed the ability of miR-944 to bind the 3´UTR of SIAH1 and PTP4A1 genes, but not PRKCA gene. Congruently, an inverse correlation between miR-944 and SIAH1 protein expression was found in breast cancer cells. Moreover, SIAH1 was upregulated in 75 % of miR-944-deficient breast tumors. Finally, SIAH1 gene silencing by RNA interference significantly impaired cell migration of breast cancer cells.

**Conclusions:**

Our results pointed out that miR-944 is a novel upstream negative regulator of SIAH1 and PTP4A1 genes and provided for the first time evidence for its functional role in migration and invasion of breast cancer cells. They also suggest that miR-944 restoration may represent a potential strategy for breast cancer therapy.

**Electronic supplementary material:**

The online version of this article (doi:10.1186/s12885-016-2470-3) contains supplementary material, which is available to authorized users.

## Background

Cancer is a major public health problem worldwide. Based on GLOBOCAN estimates, about 14.1 million new cancer cases and 8.2 million deaths occurred in 2012 around the world [1]. Notably, breast cancer is a leading cause of death in women with 1.38 million new cases diagnosed in 2008 worldwide ([2]. However, despite significant advances in screening, diagnosis, and personalized therapies, this disease still remains largely incurable. This situation is aggravated by the lack of relevant clinical molecular determinants and classifiers associated to prognostic and biological variables of patients. Therefore the search for novel biomarkers representative of the molecular features of tumors is required to better understand the mechanisms that contribute to disease progression and identify novel therapeutic targets.

MicroRNAs are evolutionary conserved small non-coding RNAs that function as negative regulators of gene expression by either inhibiting translation or inducing degradation of a set of specific messenger RNAs [3]. MicroRNAs regulate multiple physiological processes, including development, differentiation, growth, and cell death. In cancer cells, microRNAs may function either as oncogenes or tumor-suppressors (oncomiRs) [4]. Therefore, the altered expression of microRNAs may greatly contribute to the heterogeneous behavior of diverse human neoplasia and in some cases, may correlate with clinic-pathological features of tumors. Consequently, they represent novel potential prognostic biomarkers and therapeutic targets in cancer [5]. One of the most deadly hallmarks of cancer cells is their ability to metastasize to other tissues and organs [6]. This property can be promoted by a specific set of microRNAs named metastamiRs that target multiple transcripts related to cell migration [4]. It has been shown that several microRNAs target genes that drive cytoskeleton remodeling and promote tumor cell invasion [7], however, postranscriptional regulatory mechanisms involving microRNAs still remain poorly understood in cancer. Recently we performed a microRNAs profiling of breast carcinomas and found that miR-944 was significantly repressed in clinical specimens [8]. In the present study, we aimed to further investigate the biological significance of miR-944 in breast cancer. Here we identified multiple genes that are modulated by miR-944 and revealed that the cell migration-related SIAH1 and PTP4A1 genes are two novel targets of miR-944. Altogether, our data contribute for the understanding of the molecular mechanisms controlling cell migration and invasion of breast cancer cells.

## Methods

### Cell lines

Human MDA-MB-231, MCF-7, MDA-MB-453, ZR-75 and T457-D breast cancer cell lines and MCF-10A non-tumorigenic breast cells were obtained from the American Type Culture Collection and routinely grown in Dulbecco’s modified of Eagle’s medium (DMEM) supplemented with10 % fetal bovine serum and penicillin-streptomycin (50 unit/ml; Invitrogen). Cell lines were maintained at 37 °C in 5 % CO_2_.

### Tissue collection

Locally invasive breast tumors and normal tissues were provided by the Institute of Breast Diseases-FUCAM, Mexico, following the regulations approved by the FUCAM ethics committee. A written informed consent was obtained from each participant prior to release for research use. None of the enrolled patients received any antineoplastic therapy before surgery. After tumor resection, specimens were embedded in Tissue-Tek and snap frozen in liquid nitrogen at -80 °C. Pathologist confirmed the existence of at least 80 % tumor cells in clinical specimens.

### Quantitative reverse transcription and polymerase chain reaction (qRT-PCR)

The expression of miR-944 was measured by microRNA assays as implemented by manufacturer (ThermoFisher) and the comparative Ct (2 − ΔΔCt) method using an automatic baseline and a threshold of 0.2 to determine the Ct raw data. Total RNA (100 ng) of cells and tissues was obtained using the Trizol reagent (Invitrogen) and reverse transcribed using the looped-RT specific primer for miR-944, dNTPs (100 mM), reverse transcriptase MultiScribe (50 U/μl), 10X buffer, RNase inhibitor (20 U/μl) and RNase-free water. Then, retrotranscription reaction (1:15) was mixed with 10 μl master mix TaqMan (Universal PCR Master Mix, No AmpErase UNG, 2X), 7.67 μl RNase free water, and 1.0 μl PCR probe. PCR reaction was performed using a GeneAmp System 9700 (Applied Biosystems) as follows: 95 °C for 10 min, and 40 cycles at 95 °C for 15 s and 60 °C for 1 min. RNU44 was used as a control for normalization of data.

### Transfection assays

The miR-944 precursor (4464066; Life Technologies), and scramble sequence (AM17110; Life Technologies) used as negative control, were transfected into MDA-MB-231 and MCF-7 cells using siPORT amine transfection agent (Ambion, Inc., Austin, TX, USA). Briefly, pre-miR-944 was diluted in 25 μl Opti-MEM to obtain a concentration range from 50 nM to 200 nM and added to wells containing 1x10^7^ cells grown in 450 μl DMEM for 48 h. Expression of miR-944 was evaluated by qRT-PCR as described.

### Cell viability assays

MDA-MB-231 and MCF-7 cells (2x10^4^), transfected or not with miR-944 precursor (50 nM) or scramble sequence as described above, were incubated with 3-(4, 5-dimethylthiazol-2-yl)-2, 5-diphenyl tetrazolium bromide (MTT, 1 mg/ml) at 37 °C for 4 h. The formazan dye crystals were solubilized with 500 μl isopropanol, 4 mM HCl, NP-40 0.1 % for 5 min. Absorbance was measured using a spectrophotometer at 540 nm wavelength. Experiments were performed three times by triplicate and results were represented as mean ± Standard Deviation (SD).

### Cell migration and invasion assays

MDA-MB-231 and MCF-7 cells were transfected with miR-944 precursor (50 nM) or scramble as described above. Twenty-four hours postransfection, a vertical wound was traced in the cell monolayer. At 4 and 24 h, cells were fixed with 4 % paraformaldehyde and the scratched area was determined to quantify cell migration. In transwell assays, chambers (Corning) with 6.5-mm diameter and 8-μm pore size polycarbonate membrane were used. MDA-MB-231 and MCF-7 cells (1 × 10^5^) were transferred to 0.5 ml serum-free medium and placed in the upper chamber, whereas the lower chamber was loaded with 0.8 ml medium containing 10 % fetal bovine serum. The total number of cells that migrated into the lower chamber was counted after 24 h incubation at 37 °C. Cell invasiveness was evaluated using transwell chambers coated with a layer of extracellular matrix (BD Biosciences). MDA-MB-231 cells were treated with pre-miR-944 (50 nM) or scramble and 24 h postransfection, the invasive cells were quantified. Non-transfected cells were used as control. Each experiment was performed three times by triplicate and results were represented as mean ± S.D.

### Western blot analyses

Proteins obtained from breast tumors or MDA-MB-231 and MCF-7 cells transfected with miR-944 precursor (50 nM) or scramble as described above, were separated on 10 % polyacrylamide gels and transferred to PVDF membrane (Millipore). Membrane was incubated overnight at 4 °C with α-actinin-1 (sc-17829, Santa Cruz Biotechnology) or SIAH1 (ab2237 Abcam) primary antibodies, and then incubated with horseradish peroxidase–conjugated anti-mouse IgG or anti-goat IgG secondary antibodies (1:8,500, Zymed), respectively. Signal was detected and developed using the ChemiLucent (Chemicon) system.

### Indirect immunofluorescence

MDA-MB-231 and MCF-7 cells transfected with miR-944 precursor were seeded on coverslips (1x10^3^ cells/cm^2^). After 48 h, cells were rinsed with cytoskeleton buffer (10 mM MES pH 6.1, 138 mM KCl, 3 mM MgCl_2_, 2 mM EGTA, 0.32 M sucrose) at 37 °C and fixed with 3 % cytoskeleton buffer for 15 min at 37 °C to maintain the integrity of the cytoskeleton. Then, cells were permeabilized with 0.1 % Triton-X 100-CB (Sigma-Aldrich) for 5 min, blocked with 0.5 % fish skin gelatin in PBS, and incubated with phalloidin-rhodamine (0.1 μg/μl) or alpha-actinin 1 antibodies for 1 hr at room temperature (Sigma-Aldrich). Finally, slides were assembled with vectashield® mounting media (Vector) containing DAPI and cells were observed under an Olympus FluoView FV1000 Confocal Microscope with an attached MRC1024 LSCM system (Bio-Rad). Cells were imaged from top to bottom in the Z-plane; images from the basal plane of the cells were captured and stored as digital images.

### Microarrays analysis

Global gene expression analysis was done for MDA-MB-231 cells transfected with miR-944 precursor (50 nM) or scramble (30 nM) using the NimbleGen array (Roche). RNA samples were used to synthesize double-stranded labeled cDNA using SuperScript Double-Stranded cDNA Synthesis Kit (Invitrogen) and NimbleGen One-Color DNA Labeling Kit. Samples were hybridized in NimbleGen array 12x135K (12 x 135,000 features). After hybridization and washing, the processed slides were scanned using a NimbleGen MS200 Microarray Scanner. Raw data were extracted as pair files by NimbleScan software (version 2.5), background was corrected and data were normalized. The probe level files and gene summary files were produced and imported into ANAIS software (Analysis of NimbleGen Arrays Interface) for further analysis. The Student test with Varmixt package was used and raw *P* values were adjusted by the Benjamini and Yekutieli method to control the false discovery rate (FDR). Only genes with a Benjamini/Yekutieli value <0.05, and expression fold change >1.5 were considered as being differentially expressed.

### Luciferase assays

The 3´UTR region of SIAH1, PTP4A1 and PRKCA genes was cloned downstream of luciferase gene into p-miR-report vector (Ambion). Then, recombinant plasmids (2 μg) were transfected into MDA-MB-231 cells. At 24 h pre-miR-944 (50 nM) or pre-miR-negative control (scramble) were co-transfected using lipofectamine RNAi max (Invitrogen). After 24 h, firefly and Renilla luciferase activities were measured by the Dual-Glo Luciferase Assay (Promega, Charbonnieres, France) using a Fluoreskan Ascent FL (Thermo Scientific). Data were normalized with respect to Renilla activity and *p*-values for differences were determined by the two-tailed Student’s t test.

### Targeted inhibition of SIAH1

Two oligonucleotides pairs (21-23 nt length) corresponding to two short hairpin RNAs (shRNA) targeting the SIAH1 gene were designed (Additional file 1). To minimize the possibility of shRNAs off targeting effects, a nucleotide BLAST search was carried out. Each oligonucleotide pair was cloned into the pSilencer 5.1 U6 retro plasmid (ThermoFisher) and sequences were confirmed by automatic sequencing. The resulting plasmids were transfected into MDA-MB-231 cells and SIAH1 expression was evaluated by Western blot assays at 48 h post-transfection.

### Statistical analysis

Experiments were performed three times by triplicate and results were represented as mean ± S.D. One-way analysis of variance (ANOVA) followed by Tukey’s test were used to compare the differences between means. A *p* < 0.05 was considered as statistically significant.

## Results

### MiR-944 is suppressed in breast cancer cell lines and clinical tumors

**Fig. 1:**
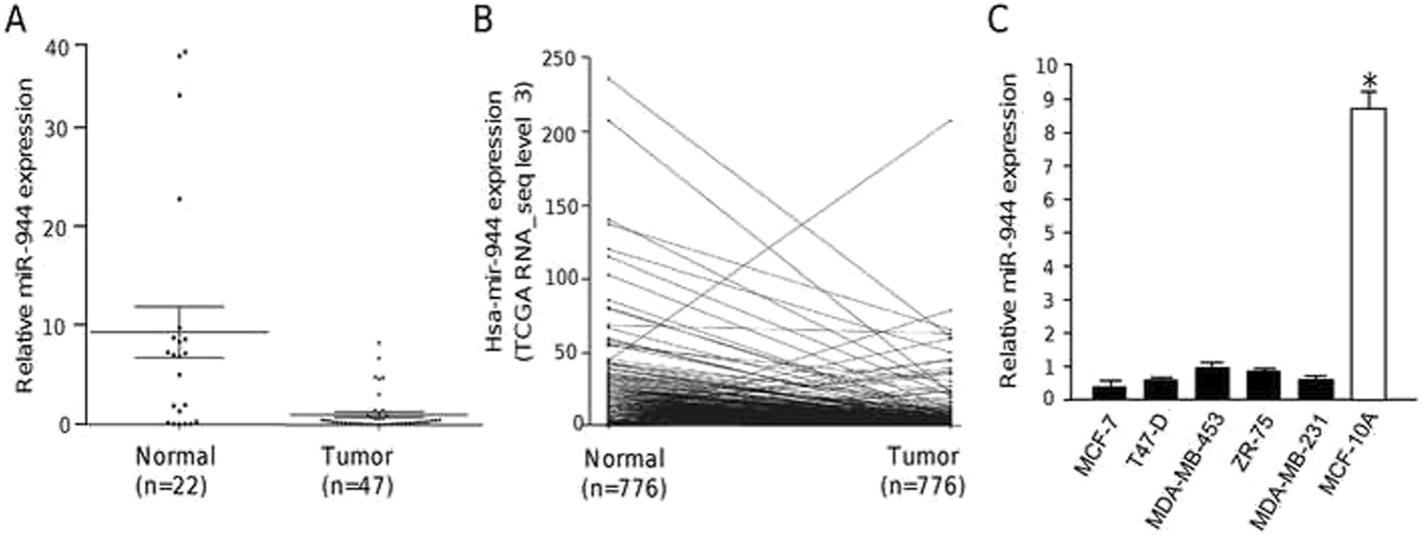
MiR-944 is suppressed in clinical tumors and breast cancer cell lines. (**a**) MiR-944 expression measured by qRT-PCR in breast normal adjacent and tumor tissues (discovery cohort). (**b**) MiR-944 expression in 776 matched normal/tumor samples from The Cancer Genome Atlas (TCGA) (validation cohort). (**c**) MiR-944 expression measured by qRT-PCR in breast cancer cell lines and MCF-10A non-tumorigenic cell line. Data were normalized with the endogenous small-nucleolar RNU44. Bars represent the mean of three independent experiments performed three times ± S.D. Asterisks indicate *p* < 0.05

In order to confirm the clinical relevance of miR-944 in breast cancer, we quantified its expression by qRT-PCR in a set of clinical specimens obtained from a cohort of 40 patients (discovery cohort) from the FUCAM institution. Clinical features of breast tumors including hormonal receptor status, tumor size, histology, clinical stage, and tumor grade are summarized in Table 1. Results indicated that miR-944 expression was significantly (*p* < 0.05) diminished in tumors in comparison with adjacent normal tissues (Fig. 1a). Our results were validated by the analysis of 776 matched normal/tumor samples at The Cancer Genome Atlas (TCGA) (validation cohort), since the average expression of miR-944 was 8.16 in normal tissues versus 3.04 in tumors (Fig. 1b). To strengthen these data, we further analyzed the TGCA data for miR-204 and miR-10b, two miRNAs that have been previously reported as down-regulated and up-regulated, respectively, in breast cancer. As expected, miR-204 was suppressed, whereas miR-10b was overexpressed in the validation cohort (Additional file 2). On the other hand, miR-944 expression was significantly lower (8 to 9-fold) in MCF-7, MDA-MBD-231, MDA-MB-45, ZR-45, and T47-D breast cancer cell lines in comparison with non-tumorigenic MCF-10A breast cell line (Fig. 1c). Taken all together, our results confirmed that miR-944 was significantly suppressed in breast tumors.

**Table 1:** Clinical features of breast tumors analyzed for miR-944 expression

Patient	Tumor size (mm)	Clinical stage	Tumor grade	HER2	ER	PR	Classification	Histological subtype
5	30	IIIB	ND	+	-	-	Her2+	Infiltrating ductal carcinoma
13	15	I	ND	-	+	+	Luminal A	*In situ* papillary carcinoma
24	25	IIA	ND	+	-	-	Her2+	Infiltrating papillary carcinoma
50	25	IIA	2	-	+	+	Luminal A	Infiltrating ductal carcinoma
55	20	I	3	+	-	-	Her2+	Infiltrating ductal carcinoma
58	35	IIA	2	+	-	-	Her2+	*In situ* ductal carcinoma
59	ND	ND	2	-	+	+	Luminal A	Infiltrating ductal carcinoma
71	19	IIIA	2	-	+	-	Luminal A	Infiltrating ductal carcinoma
73	20	IIIA	ND	+	+	+	Luminal B	Infiltrating ductal carcinoma
74	35	IIB	ND	-	-	+	ND	Infiltrating ductal carcinoma
79	20	IIIB	2	-	+	-	Luminal A	Infiltrating ductal carcinoma
80	25	IIA	3	-	-	-	Triple negtive	Infiltrating ductal carcinoma
81	25	IIA	3	-	-	+	ND	Infiltrating ductal carcinoma
82	25	IIB	ND	+	-	-	Luminal A	Infiltrating ductal carcinoma
97	47	IIIA	3	-	-	-	Triple negative	Infiltrating ductal carcinoma
98	20	I	ND	-	+	-	Luminal A	Infiltrating ductal carcinoma
106	16	I	ND	-	+	+	Luminal A	Infiltrating ductal carcinoma
107	20	I	2	-	+	+	Luminal A	Infiltrating ductal carcinoma
110	25	IIA	2	-	+	-	Luminal A	Infiltrating ductal carcinoma
113	17	I	1	-	+	+	Luminal A	*In situ* lobular carcinoma
122	16	IIA	3	-	-	-	Triple negative	Infiltrating ductal carcinoma
125	25	IIB	2	-	+	-	Luminal A	Infiltrating ductal carcinoma
128	22	IIA	ND	-	+	+	Luminal	Infiltrating mucinous carcinoma
129	13	IIA	ND	-	+	+	Luminal A	Infiltrating ductal carcinoma
139	30	IIB	3	-	-	-	Triple negative	Infiltrating ductal carcinoma
142	18	IIA	ND	-	-	+	ND	Infiltrating lobular carcinoma
144	35	IIB	3	-	-	-	Triple negative	Infiltrating ductal carcinoma
146	65	IIIB	3	-	-	-	Triple negative	Infiltrating ductal carcinoma
149	30	IIB	2	-	+	-	Luminal A	Infiltrating ductal carcinoma
150	10	0	1	-	+	+	Luminal A	*In situ* ductal carcinoma
168	40	IIB	2	-	-	-	Triple negative	Infiltrating ductal carcinoma
186	45	IIB	2	-	+	+	Luminal A	Infiltrating ductal carcinoma
189	40	IIB	ND	-	-	-	Triple negative	Infiltrating medular carcinoma
2b	55	IIIA	ND	-	-	-	Triple negative	Infiltrating lobular carcinoma
3b	10	I	3	-	-	-	Triple negative	Infiltrating ductal carcinoma
4b	11	I	1	-	-	-	Triple negative	Infiltrating ductal carcinoma
7b	ND	ND	ND	-	-	-	Triple negative	Infiltrating ductal carcinoma
8b	17	I	3	-	-	-	Triple negative	Infiltrating ductal carcinoma
9b	30	IIA	2	-	-	-	Triple negative	Infiltrating ductal carcinoma
10b	27	IIB	3	-	-	-	Triple negative	Infiltrating ductal carcinoma

ND, No determined; ER, Estrogen receptor; PR, Progesterone receptor; HER2, Human epidermal growth factor receptor 2

### MiR-944 inhibits cell migration and invasion

**Fig. 2:**
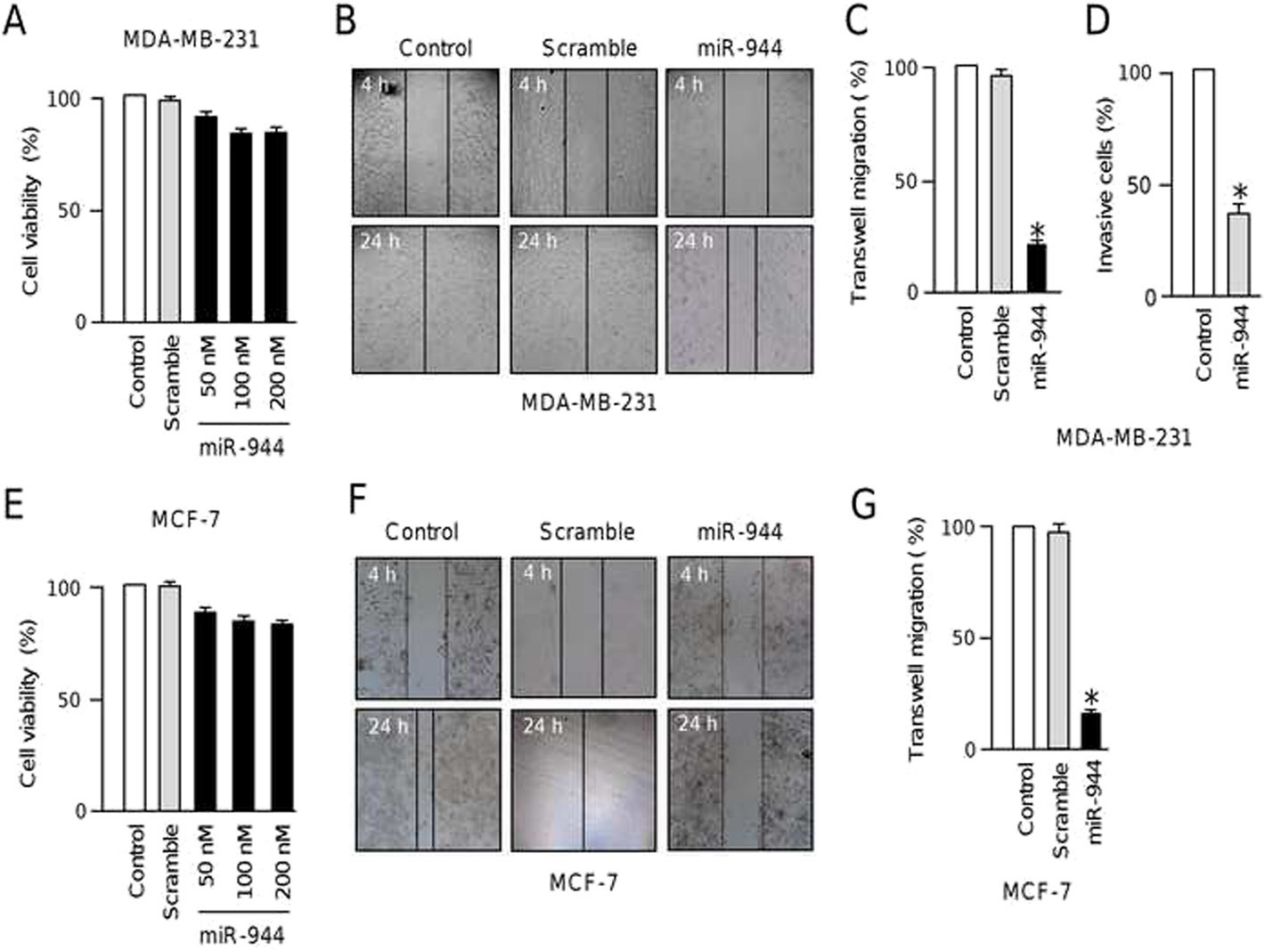
MiR-944 suppresses cell migration and invasion. (**a** and **e**) MTT cell viability assays of MDA-MB-231 (a) and MCF-7 (e) cells transfected with miR-944 precursor (50 nM to 200 nM). (**b** and **f**) Scratch/wound-healing assays of MDA-MB-231 (b) and MCF-7 (f) cells monolayers treated with miR-944 precursor (50 nM). (**c** and **g**) Transwell assays of MDA-MB-231 (c) and MCF-7 (g) cells transfected with miR-944 precursor (50 nM). (**d**) Matrigel invasion assays of MDA-MB-231 cells transfected with miR-944 precursor (50 nM). Non-transfected cells were used as controls. Bars represent the mean of three independent experiments performed three times ± S.D. Asterisks indicate *p* < 0.05

To define the functions of miR-944 we restored its expression using RNA mimics in triple negative MDA-MB-231 (highly metastatic) and oestrogen responsive MCF-7 (poorly invasive) breast cancer cells (Additional file 3). First, the effect of diverse concentrations of miR-944 precursor on cell viability was evaluated by MTT assays. Results showed minimal changes (less than 5 %) in cell viability of MDA-MB-231 transfected with 50 nM miR-944 precursor in comparison with scramble transfected and non-transfected controls. Using 100 nM and 200 nM miR-944 precursor, we observed a 10 % reduction on cell viability relative to controls (Fig. 2a). Similar results were obtained in MCF-7 cells (Fig. 2e). Then, we performed scratch/wound-healing assays in both breast cancer cell lines to evaluate the contribution of miR-944 in tumor cell migration. Data indicated that cell monolayers restoration was delayed in both MDA-MB-231 and MCF-7 cells transfected with miR-944 precursor (50 nM) when compared with non-treated and scramble-transfected cells at 24 h (Fig. 2b and f). In addition, transwell chamber assays showed that the number of migratory cells was significantly (*p* < 0.05) reduced in MDA-MB-231 (4-fold) and MCF-7 (8-fold) cells that ectopically express miR-944 (Fig. 2c and g) in comparison with control cells. Moreover, miR-944 significantly (*p* < 0.05) inhibited the ability of metastatic MDA-MB-231 cells to invade matrigel *in vitro* (Fig. 2d).

### MiR-944 alters cytoskeleton organization

**Fig. 3:**
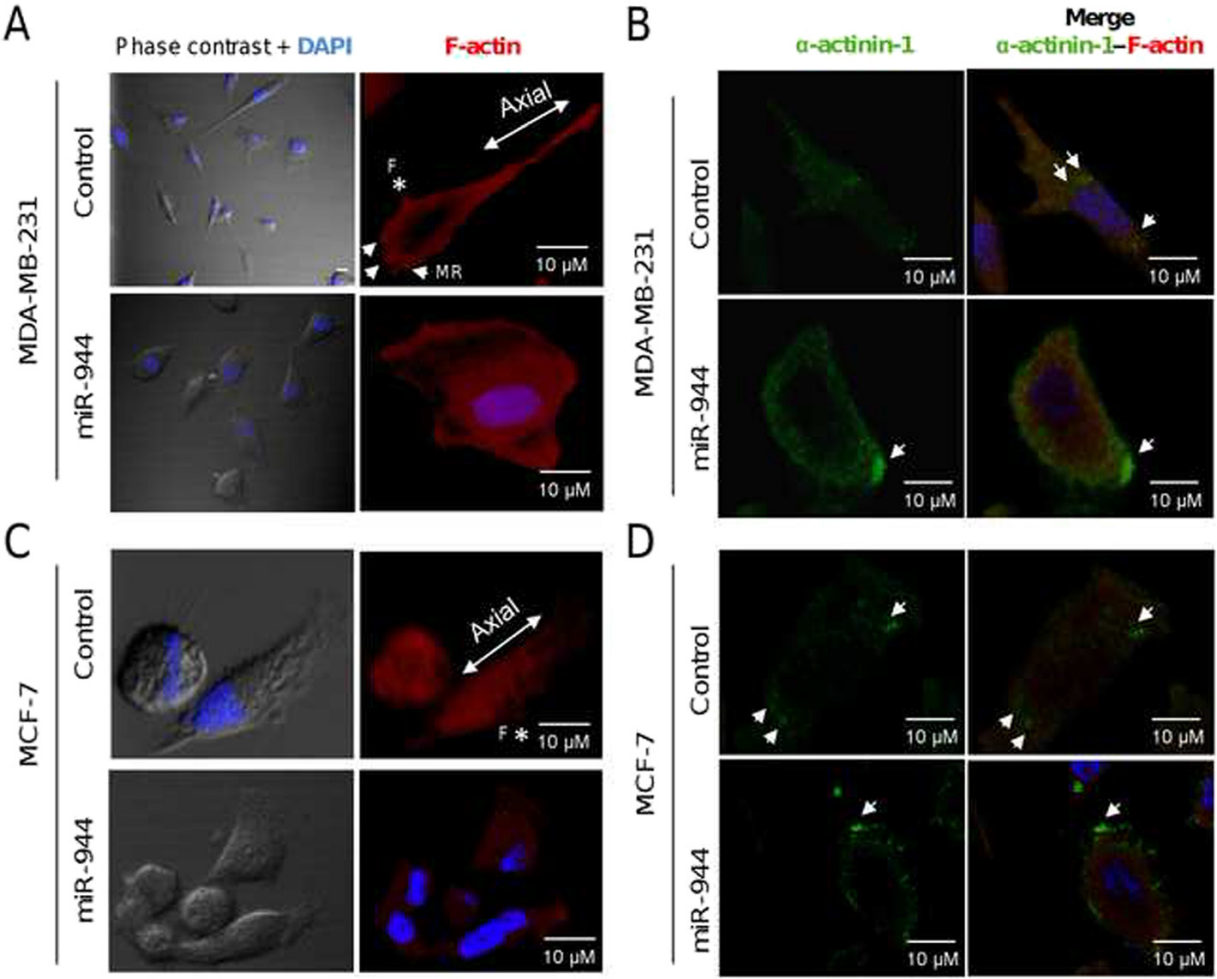
MiR-944 alters cytoskeleton and focal adhesions. MDA-MB-231 and MCF-7 cells were treated for indirect immunofluorescence with rhodamine phalloidin to visualize F-actin (red) or with α-actinin1 antibody labeled with FITC (green). Nuclei were counterstained with DAPI (blue). (**a**) Phase contrast and immunofluorescence images show actin organization in non-transfected (control) and miR-944 expressing MDA-MB-231 (top panels) and (**c**) MCF-7 cells (bottom panels). Arrowheads indicate representative actin-rich membrane ruffling (MR); asterisk indicates filopodia (*f*). (**b**) Representative x-z confocal images of α-actinin-1 (green) and F-actin (red) organization in MDA-MB-231 (top panels) and (**d**) MCF-7 cells (bottom panels) non-transfected (control) or transfected with miR-944 precursor

As cell migration may involve the coordinated expression and association of proteins driving the epithelial-mesenchymal transition (EMT), cytoskeleton organization and reinforcement of focal adhesions, we decided to determine if miR-944 contributes to these cellular processes. We first analyzed the expression of proteins modulating the EMT, including SIP1, ZEB1 and BMP2, by Western blot assays. Results showed no significant changes in the expression of these proteins in miR-944 expressing cells (data not shown). Then, we examined the organization of cytoskeleton in MDA-MB-231 and MCF-7 cells by analyzing the distribution of F-actin labeled with rhodamine-phalloidin using confocal microscopy. As α-actinin-1 is an actin-crosslinking protein that reinforces focal adhesions its subcellular distribution was also examined. As depicted in Fig. 3a (upper panels), MDA-MB-231 control cells were featured by an axial F-actin cytoskeleton organization, and the presence of structures, such as membrane ruffles (MR) and filopodia (F) associated to a migrating phenotype were evident. Interestingly, the ectopic expression of miR-944 induced a dramatic effect on overall cell morphology since spread area was increased (Fig. 3a bottom panel). Moreover, F-actin was redistributed in a radial mode towards the periphery of the cell, as well as in the central zone; and the membrane ruffles and filopodia structures were lost. Based on these morphological differences, we next analyzed the strengthening of adhesion-related structures. MDA-MB-231 cells transfected with miR-944 precursor exhibited a robust signal of α-actinin-1 and an increase in the number of contact points with F-actin in multiple points of cell body, indicative of the reinforcement of focal adhesions. Remarkably, these cells displayed enrichment in α-actinin-1-rich blebs at the rear end of the cell suggesting a strong adhesive process (Fig. 3b bottom panel). Likewise, restoration of miR-944 expression in MCF-7 cells induced changes in actin cytoskeleton organization and loss of the axial pattern in a similar manner as in MDA-MB-231 cells (Fig. 3c bottom panel). In addition, α-actinin-1 was redistributed and accumulated in focal points at the end or front of cells indicative of focal adhesions formation (Fig. 3d, bottom panel), although in a less extend in comparison with MDA-MB-231 cells transfected with miR-944 precursor.

### MiR-944 modulates genes involved in cell adhesion and migration

In order to identify potential target genes of miR-944 that may explain the phenotypic changes described above, we carried out a transcriptional profiling of MDA-MB-231 cells that ectopically express miR-944 using DNA microarrays. Results evidenced that 1197 genes were significantly downregulated and 144 were upregulated (fold change >1.5; Additional file 4). Some of these modulated genes are well known cancer-related genes including MAPK1, IGF1R, SIAH1, PRKCA, RAC1, NOTCH2, MMP14, PAK1 and PTP4A1, among others. Classification of the set of repressed genes based on GO categories showed that 15 genes are involved in cell migration and invasion processes (Table 2).

**Table 2:** Suppressed genes in miR-944 transfected cells with roles in cell migration and invasion

^a^Gene symbol	^b^Protein name	Fold change	Associated function in cancer	miR-944 binding sites^c^
NEK2	Serine/threonine-protein kinase Nek2 (Never in mitosis A-related kinase 2)	-3.09	Nek2 is up-regulated in pre-invasive *in situ* ductal and invasive breast carcinomas	0
ADAM28	Disintegrin and metalloproteinase domain-containing protein 28	-3.05	ADAM28 is overexpressed in lymph node metastasis in lung carcinomas	0
PAK1	Serine/threonine-protein kinase p21-activated kinase1	-3.03	PAK1 induces colorectal cancer metastasis by ERK activation and FAK-Ser901 phosphorylation	0
FGFR2	Fibroblast growth factor receptor 2	-3.01	Overexpression of FGFR2, a transforming oncogene in human mammary epithelial cells, leads to invasive phenotype	0
RAC1	Ras-related C3 botulinum toxin substrate 1	-2.98	RAC1 activation mediates Twist1-induced cancer cell migration	0
ANXA7	Annexin A7	-2.67	Decreased ANXA7 expression is associated with high invasive potential in multiple tumors	0
NCOA4	Nuclear receptor coactivator 4	-2.34	NCOA4 (ARA70) promotes cell growth and invasion in prostate cancer	0
MMP14	matrix metallopeptidase 14	-2.3	MMP14 controls invasiveness of aggressive breast tumours, and is associated with clinical outcome	1
PLCB2	1-phosphatidylinositol 4,5-bisphosphate phosphodiesterase beta-2	-2.23	Promotes mitosis and migration of human breast cancer-derived cells	0
PRKCA	Protein kinase C, alpha	-1.96	PRKCA regulate Ets1 in invasive breast cancer	1
SIAH1	E3-ubiquitin protein ligase	-1.90	Promotes migration and invasion of glioma cells by regulating HIF-1 under hypoxia. Impairs tumor growth and metastasis inbreast cancer	1
PTP4A1	Protein tyrosine phosphatase type IVA, member 1	-1.80	PTP4A1 is related to the lymph node metastasis of colonic adenocarcinoma. Promotes cell motility, invasion and metastasis of ovarian and lung cancer cells.	1
DEK	DEK	-1.58	DEK oncogene regulates motility and invasion in breast cancer	0
NOTCH2	Neurogenic locus notch homolog protein 2	-1.58	Plays a role in invasive breast cancer	0
TRIM32	E3 ubiquitin-protein ligase TRIM32 (Tripartite motifcontaining 32)	-1.53	TRIM32 oncogene promotes tumor growth, metastasis, and resistance to anticancer drugs via degradation of Ablinteractor 2	1

^a^GenBank databases. ^b^Uniprot database (Recommended name). ^c^Predicted by TargetScan

### MiR-944 targets SIAH1 and PTP4A1 genes

**Fig. 4:**
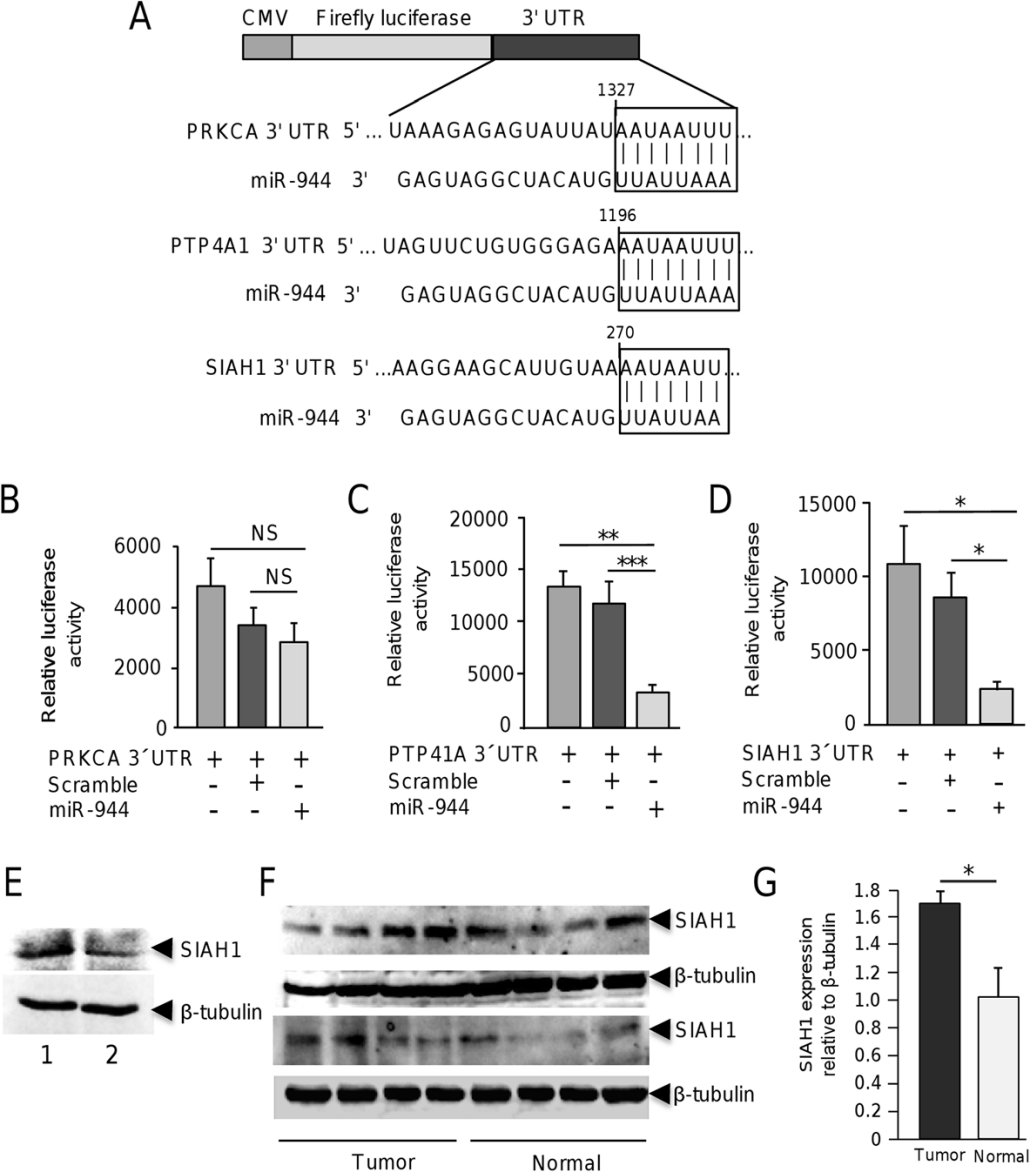
SIAH1 and PTP41A genes are miR-944 targets. (**a**) Schematic representation indicating the 3´UTR sequence of PRKCA, PTP4A1 and SIAH1 genes cloned in pmiR-report vector. Boxes indicate the miR-944 binding sites in target genes. (**b**, **c**, **d**) Luciferase reporter assays. MDA-MB-231 cells were co-transfected with miR-944 (or scramble as control) and pmiR-LUC-PRKCA-3´UTR (**b**), pmiR-LUC-PTP4A1-3´UTR (**c**) or pmiR-LUC-SIAH1-3´UTR (**d**) plasmids and relative luciferase activity was measured as described in methods. Results are expressed in light units. Bars represent the mean of three independent experiments performed three times ± S.D. (**e**) Immunodetection of SIAH1 by Western blot assays in MDA-MB-231 cells. Lane 1, MDA-MB-231 control cells; lane 2, MDA-MB-231 cells transfected with miR-944. (**f**) Immunodetection of SIAH1 in breast tumors and normal mammary tissues. β-tubulin was used as internal control. (**g**) Densitometry analysis of immunodetected bands in F. Pixels corresponding to β-tubulin were used to normalize SIAH1 expression. NS, non- significant. *p < 0.05; **p < 0.01; ***p < 0.001

Data from DNA microarrays led us to the identification of potential new target genes for miR-944. Surprisingly, no genes involved in EMT and focal adhesions were found as directly modulated, thus we focused on genes involved in cell migration and cytoskeleton dynamics. Interestingly, the cell migration-related SIAH1, PTP4A1 (also known as PRL-1), and PRKCA genes were repressed after transfection of miR-944 precursor. These genes are key regulators of cell migration and cancer progression in diverse types of cancer [9–11]. Therefore, we investigated if SIAH1, PTP4A1 and PRKCA genes are direct targets of miR-944 using luciferase reporter assays. We identified the complementary site for miR-944 in the 3´UTR sequence of each gene and cloned it downstream of the luciferase coding region in the pmiR-report vector (Fig. 4a). Results showed that forced expression of miR-944 and co-transfection of pmiR-LUC-PRKCA-3´UTR did not result in significant differences in luciferase activity (Fig. 4b). In contrast, the co-transfection of miR-944 and pmiR-LUC-SIAH1-3´UTR or pmiR-LUC-PTP4A1-3´UTR plasmids significantly reduced the luciferase activity (*p* < 0.001 and *p* < 0.05, respectively) in comparison with controls (Fig. 4c and d). Because of its relevant role in migration of cancer cells we next focused in the analysis of the SIAH1 protein. Western blot assays revealed that SIAH1 protein levels were reduced in MDA-MB-231 cells transfected with miR-944 in comparison to non-transfected control cells (Fig. 4e). Congruently, the expression of SIAH1 was significantly increased in 53 % of miR-944 deficient breast tumors in comparison with normal adjacent tissues (Fig. 4f and 4g).

### Knockdown of SIAH1 impairs cell migration

**Fig. 5:**
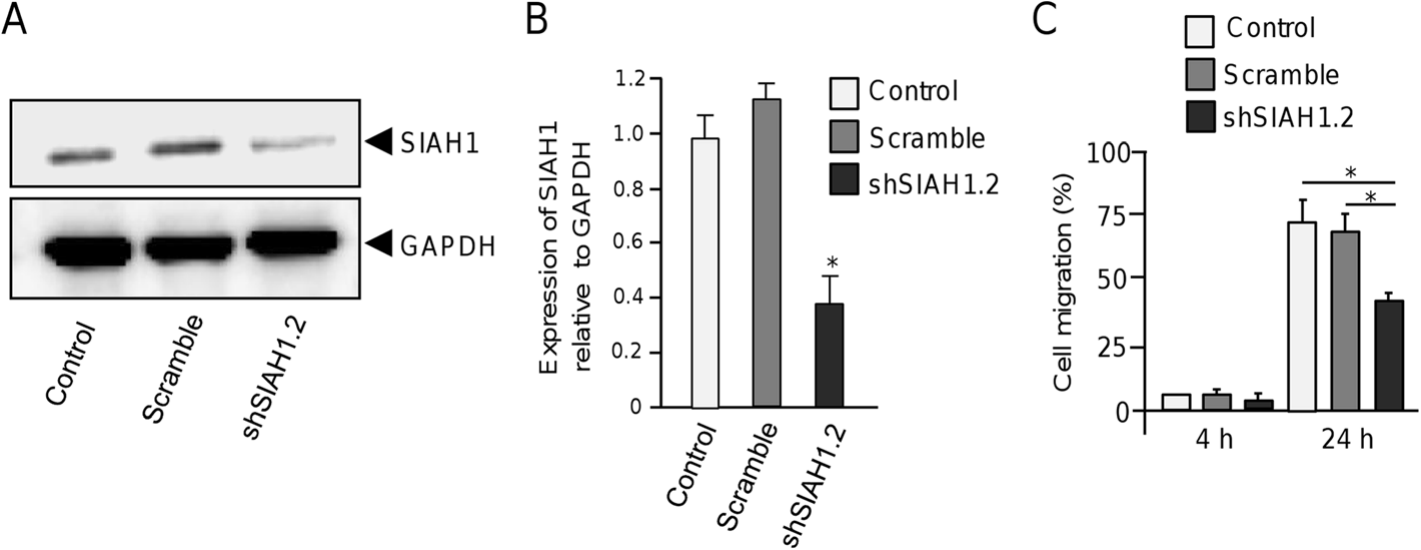
SIAH1 silencing inhibits cell migration of breast cancer cells. (**a**) Western blot assays for SIAH1 knock-down in MDA-MB-231 cells using shSIAH1.2 interfering sequence. Scramble sequence was transfected as negative control. GAPDH was used as internal loading control. (**b**) Densitometric analysis of immunodetected bands in panel A. (**c**) Quantification of scratch/wound healing assays in non-transfected control, scramble transfected and SIAH1-deficient cells. Data represents the mean of three independent assays ± SD. (*p < 0.05)

To determine if targeted inhibition of SIAH1 affects cell migration we proceeded to knock-down its expression using RNA interference. Two specific short hairpin RNAs (dubbed as shSIAH1.1 and shSIAH1.2) targeting the human *SIAH1* gene were designed and cloned into the pSilencer vector (Additional file 1). Both constructs were individually introduced into MDA-MB-231 cells and SIAH1 expression was analyzed by Western blot 48 h after transfection. Results showed that shSIAH1.2 sequence down-regulated the SIAH1 expression (Fig. 5a), whereas no significant effect was observed with shSIAH1.1 interfering sequence (data not shown). The expression of GADPH used as a control, did not show significant changes between treatments. Densitometric analysis of immunodetected bands showed that silencing induced by shSIAH1.2 construct was effective since this sequence suppressed SIAH1 expression by 42 % (Fig. 5b). The effect of SIAH1 silencing in cell migration was evaluated in MDA-MB-231 cells by scratch/wound-healing assays. Results showed that restoration of cell monolayers was significantly (*p* > 0.05) delayed in SIAH1-deficient cells when compared with scramble-transfected cells and non-treated control cells at 24 h (Fig. 5c).

## Discussion

One of the most devastating hallmarks in breast cancer is represented by metastasis that is related to alterations in cell adhesion and migration. Evidence is now emerging indicating that microRNAs might constitute a regulatory event in cell migration [12]. Here, we described the biological significance and the effects of miR-944 dysregulation on cell migration in human breast cancer cells. Interestingly, miR-944 gene is located in the intron of the tumor suppressor protein p63 gene, which is a transcription factor frequently suppressed in breast cancer [13]. A feedback between p63 and several microRNAs has been observed in cancer. Tucci et al. [14] reported that loss of p63 and its miR-205 target results in increased cell migration and metastasis in prostate cancer. In order to elucidate the relevance of miR-944 in breast cancer, we first characterized MDA-MB-231 and MCF-7 cells that ectopically express miR-944. According to wound healing, transwell, and matrigel experiments, the restoration of miR-944 expression resulted in a significant reduction in cell migration and invasion. Intriguingly, impaired cell migration was featured by an increased association of α-actinin-1 with F-actin cytoskeleton on focal adhesion points, and loss of membrane ruffling and filopodia. These data suggested that miR-944 plays a significant role in the control of breast cancer cell morphology as cells lost the elongated shape associated with motile and mesenchymal cells, and adopted a spread, and unpolarized shape. During the preparation of this manuscript, an interesting study about miR-944 in cervical cancer was published. Xie et al. [15] showed that miR-944 is overexpressed in human cervical cancer cells and promotes cell proliferation, migration and invasion, while it has no effect on apoptosis. These, and our data, reflect the heterogeneous nature of tumors and indicate that miR-944 functions are tumor-specific.

In order to identify genes modulated by miR-944 that could be relevant in the underlying mechanism of cell migration, we defined the transcriptional profile of MDA-MB-231 cells that ectopically express miR-944. Bioinformatics analyses of modulated genes identified novel potential targets involved in cellular pathways related to cytoskeletal remodeling and cell migration. One interesting gene was SIAH, an E3 ubiquitin-protein ligase that belongs to a family of RING-domain proteins, including the ubiquitin ligases targeting proteins for proteasomal degradation. In diverse types of cancer, SIAH1 has a dual function in RAS, estrogen, DNA-damage, and hypoxia pathways therefore it is considered as an attractive anticancer drug target [16]. However, the proteosome inhibitor bortezomib used in clinical practice inhibits all the proteosome-mediated proteolysis without specificity causing systemic toxicity and resistance; thus the search for more specific E3 ubiquitin ligases is needed [17] In mouse models, the inhibition of SIAH proteins impairs tumor growth and metastasis of breast tumors [18]. Moreover, a number of studies have linked SIAH1 expression with disease progression in human cancer [19]. However, these studies reported opposite results indicating that SIAH1 may function both as an oncogene or a tumor suppressor depending on tumor type. Behling et al. [20] reported that SIAH levels were significantly increased in ductal carcinoma *in situ* compared with normal tissues. Moreover, tumors from patients with disease recurrence had higher SIAH expression than those from patients without recurrence. In patients with hepatocellular carcinoma (HCC), nuclear accumulation of SIAH1 was correlated with carcinogenesis, tumor proliferation and migration [21]. Furthermore, reduction of SIAH1 expression levels using RNA interference in HCC decreased tumor cell viability [22]. In our study, we observed that SIAH1 expression was decreased in almost half of breast tumors analyzed, which agreed with previous studies. Importantly, we demonstrated that miR-944 was able to down-regulate SIAH1 *in vitro*. Moreover, miR-944 and SIAH1 expression showed an inverse correlation in breast tumors. In addition, targeted silencing of SIAH1 using shRNAs confirmed the role of this protein in breast cancer cells migration. These findings suggested that the effects of miR-944 in cell migration may occur, at least in part, through targeting of SIAH1.

Another validated target of miR-944 in this study was the protein tyrosine phosphatase 4A1 (PTP4A1, also known as PRL-1). Interestingly, it was reported that PRL-1 promotes cell migration and invasion by regulating filamentous actin dynamics of A549 lung cancer cells [23]. PRL-1 also decreased the expression of focal adhesion proteins. Moreover, reduction in PRL-1 was associated to decrease cell membrane protrusions with a reduction in actin fiber extensions, which could reflect reduced adhesion turnover [24]. Tumor migration and metastasis are dynamic cellular processes that continuously exploit phospho-relay signaling systems. Overexpression of PRL-1 has been identified in pancreatic cancer cell lines [25]. Zheng et al. [26] demonstrated that PRL-1 promotes cell motility, invasion, and metastasis in ovarian cells. In addition, PRL-1 induced metastatic tumor formation in mice. In light of these findings, PRL-1 has been considered as a therapeutic target in cancer [27]. Here, we showed that miR-944 was able to bind the 3´UTR of PTP4A1 downregulating its expression at mRNA level. Moreover, miR-944 expressing cells exhibited morphological changes associated to alterations in actin cytoskeleton and focal adhesions that were similar to those describe in PLR-1-deficient cells. In summary, our findings showed for the first time that miR-944 expression was dramatically suppressed in breast cancer cell lines and tumors independently of hormonal status or metastatic potential. Thus, we cannot in the present study establish a correlation between the low expression of miR-944, the metastatic potential and hormonal receptors expression. The effects of miR-944 in cell migration inhibition may occur, at least in part, through targeting of SIAH1 and PTP4A1. In addition, our data pointed out that knockdown of gene expression by miR-944 could represent a molecular tool to specifically inhibit relevant druggable targets such as SIAH1 and PTP4A1 in breast cancer.

## Conclusions

Our data provided evidences about the role of miR-944 as a novel upstream negative regulator of PTP4A1 and SIAH1 and contributed for the understanding of the molecular mechanisms controlling cell migration and invasion in breast cancer. This study also suggested that miR-944 restoration may represent a potential novel strategy for breast cancer therapy.

## Abbreviations

DMEM, Dulbecco’s modified of Eagle’s medium; EMT, Epithelial-mesenchymal transition; MR, Membrane ruffles; PRKCA, Protein kinase C alpha; PTPA41, Protein tyrosine phosphatase 4A1; SIAH1 E3, ubiquitin-protein ligase

## Additional files

Additional file 1: Oligonucleotides sequences used to silencing SIAH1 gene expression. (PDF 6 kb) Additional file 2: Comparative expression of miR-944 vs miR-204 and miR-944 vs miR-10b in TGCA validation cohort. (PDF 818 kb) Additional file 3: Taqman microRNA assay for miR-944 expression in MDA-MB-231 breast cancer cell line. (PDF 20 kb) Additional file 4: Raw data obtained from gene expression studies using DNA microarrays. (XLS 2.36 mb)
