# Supplementary material for: Suppression of cell migration is promoted by miR-944 through targeting of SIAH1 and PTP4A1 in breast cancer cells
Source: BMC Cancer. 2016 Jul 4;16:379. doi: 10.1186/s12885-016-2470-3 (PMC4932667; doi:10.1186/s12885-016-2470-3)
Supplement: Additional file 1: — Oligonucleotides sequences used to silencing SIAH1 gene expression. (PDF 6 kb) [file 12885_2016_2470_MOESM1_ESM.pdf]

Name	Sequence 5´-3´	Target región
Sh-SIAH1.1S	GATCCGCTATGGAGAAAGTGGCTAATTTCAAGAGAAT TAGCCACTTTCTCCATAGCTTTTTTGGAAA	373-394 GCTATGGAGAAAGTGGCT AAT
Sh-SIAH1.1AS	AGCTTTTCCAAAAAAGCTATGGAGAAAGTGGCTAATT CTCTTGAAATTAGCCACTTTCTCCATAGCG	
Sh-SIAH1.2S	GATCCGCTAGACACATGAAGGTAAATTCAAGAGATTT ACCTTCATGTGTCTAGCTTTTTTGGAAA	1069-1090 GCTAGACACATGAAGGTA AAT
Sh-SIAH1.2AS	AGCTTTTCCAAAAAAGCTAGACACATGAAGGTAAATC TCTTGAATTTACCTTCATGTGTCTAGCG	

Additional file 1. Oligonucleotides sequences used to silencing SIAH1 gene expression
