# Supplementary figures and images for: Suppression of cell migration is promoted by miR-944 through targeting of SIAH1 and PTP4A1 in breast cancer cells
Source: BMC Cancer. 2016 Jul 4;16:379. doi: 10.1186/s12885-016-2470-3 (PMC4932667; doi:10.1186/s12885-016-2470-3)

Additional file 2. miR-944 vs miR-204 and miR-944 vs miR-10b expression in TCGA validation cohort.

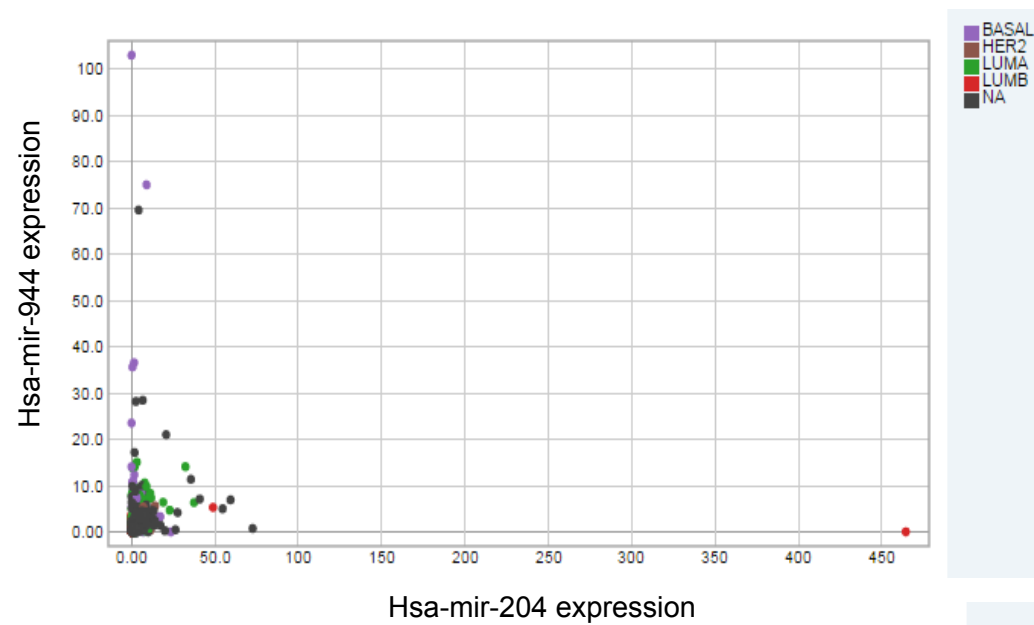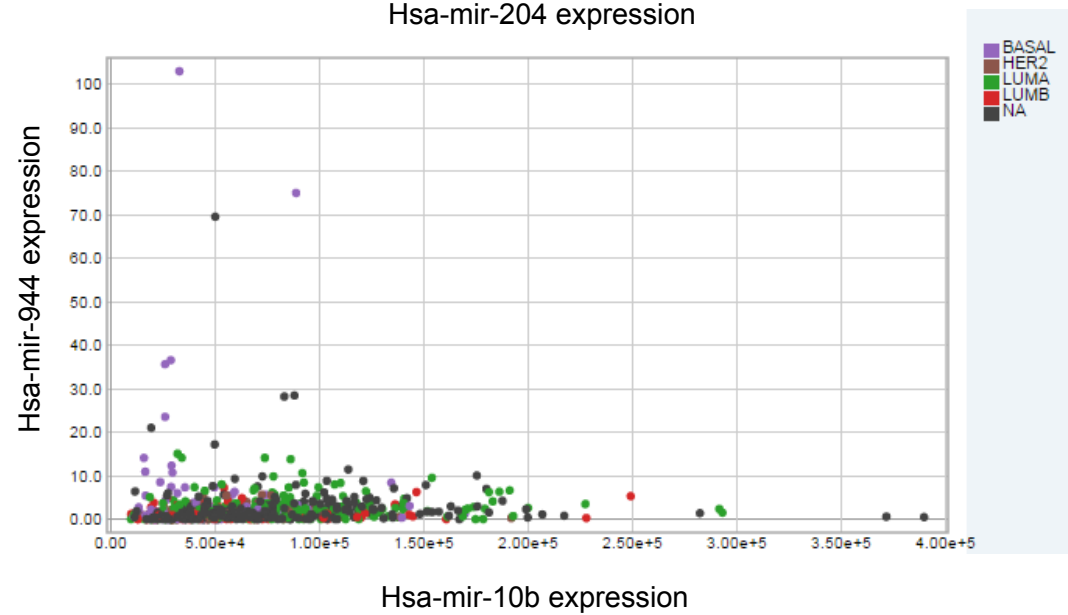

Supplement: Additional file 2: — Comparative expression of miR-944 vs miR-204 and miR-944 vs miR-10b in TGCA validation cohort. (PDF 818 kb) [file 12885_2016_2470_MOESM2_ESM.pdf]

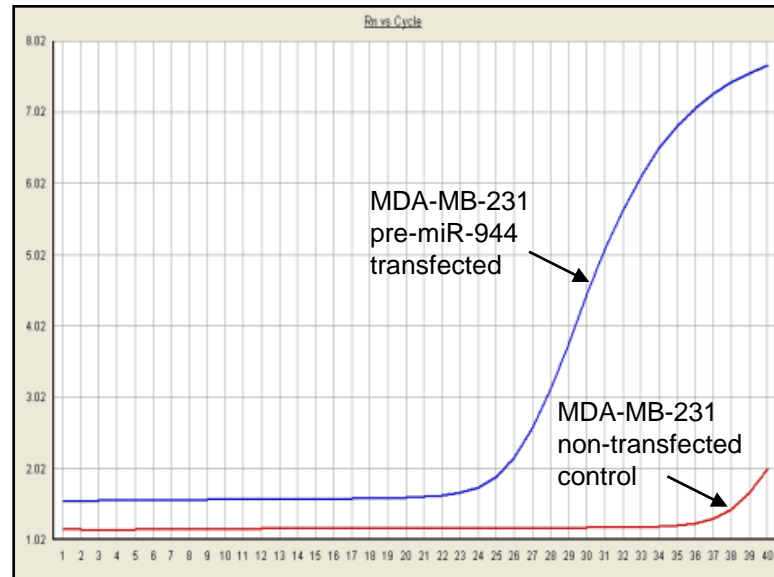

Additional file 3. Taqman microRNA assay for miR-944 expression in MDA-MB-231 breast cancer cell line.

Supplement: Additional file 3: — Taqman microRNA assay for miR-944 expression in MDA-MB-231 breast cancer cell line. (PDF 20 kb) [file 12885_2016_2470_MOESM3_ESM.pdf]
